# EndoSCell: A portable intraoperative histopathological microscopy device for enhanced glioma resection and prognosis in animal models

**DOI:** 10.1063/5.0272531

**Published:** 2025-10-02

**Authors:** Yu-Chien Lin, Ke-Jun He, Ming-Yang Jiang, Tao-Chieh Yang, Yuan-Yun Tseng, Nu Zhang, Ren-Jei Chung

**Affiliations:** 1 Department of Chemical Engineering and Biotechnology, National Taipei University of Technology, Taipei 10608, Taiwan; 2 School of Materials Science and Engineering, Nanyang Technological University, 50 Nanyang Avenue, Singapore 639798, Singapore; 3 The first affiliated hospital, Sun Yat-Sen University, No. 58 Zhongshan Er Road, Guangzhou 510080, Guangdong Province, People's Republic of China; 4 Department of Neurosurgery, School of Medicine, Chung Shan Medical University Hospital, Taichung 40201, Taiwan; 5 School of Medicine, Chung Shan Medical University, Taichung 40201, Taiwan; 6 Department of Neurosurgery, New Taipei Municipal Tu-Cheng Hospital (Built and operated by Chang Gung Medical Foundation), New Taipei City 236017, Taiwan; 7 High-value Biomaterials Research and Commercialization Center, National Taipei University of Technology (Taipei Tech), Taipei 10608, Taiwan

## Abstract

Safe maximal resection remains a critical goal and prognostic factor in glioma treatment. Neurosurgeons face significant challenges in accurately identifying the true extent of glioma during surgery due to its highly infiltrative nature. Currently, there are no tools available that allow for real-time, *in vivo* observation of surgical margins. This study introduces EndoSCell, a portable, anti-shake intraoperative histopathological microscopy device designed to overcome these limitations. EndoSCell is resistant to vibrations typical in the operating room and can be used to accurately identify the margins of malignant gliomas (MG) during surgery. The effectiveness of EndoSCell was assessed through *ex vivo* neuropathological analysis of tumor and brain tissues from glioblastoma patients, as well as *in vivo* analysis using 9L glioma and patient-derived tumor xenograft models. Craniectomies were performed on 9L orthotopic glioma-xenograft rat models with or without EndoSCell assistance, conducted by two different neurosurgeons. EndoSCell successfully demarcated the invasive margins of tumor xenografts. Postoperative magnetic resonance imaging and pathological examinations confirmed its ability to delineate tumor margins effectively. Both *ex vivo* and *in vivo* analyses showed that EndoSCell distinguishes tumor cells from normal brain cells with high precision. These findings demonstrate that EndoSCell-guided surgery not only achieves negative margins but also significantly reduces tumor recurrence, substantially improving survival rates (86% vs 20%; *P* < 0.001) in MG-bearing rats. This technology enables more precise and complete tumor resections, thereby improving progression-free survival in glioma-xenograft models.

## INTRODUCTION

Gliomas, originating from glial cells, are diffusely distributed throughout the central nervous system and extensively intermingle with the surrounding brain parenchyma.^1^ Astrocytomas (WHO grades 2 and 3), oligodendrogliomas (grades 2 and 3), and glioblastomas (GBMs, grade 4) are classified as diffusely invasive brain tumors.^2–4^ Despite over four decades of research into various agents and multimodal treatments, as well as advancements in targeted molecular marker diagnostics for early prediction,^5^ the median survival rate of patients with GBMs has not significantly increased.^6,7^ Safe maximal resection is an independent predictor and the best prognostic factor for patients diagnosed with GBM; prognosis is maximally improved with complete resection.^8,9^ However, complete resection is attainable in only 36% of surgeries, and the overall 5-year survival rate remains below 10%, with a median overall survival of 14.6 months, even when adjuvant radiotherapy and temozolomide are administered.^10,11^ The extent of resection (EOR) has been associated with greater overall survival and longer progression-free survival. However, EOR is highly impeded by the infiltrative nature of the tumor, its location, and the surgeon's skill.^12,13^ The infiltration of brain tumors presents a challenge for surgeons in distinguishing tumor margins, as they frequently appear invisible or indistinct during surgery.^14,15^ Incomplete resection results in residual tumors or microscopic satellite foci remaining within the resection cavity, which are primary factors contributing to early recurrence and unfavorable prognosis.^8,11^ Recent evidence indicates that intraoperative visualization of malignant glioma (MG) tissue using fluorescence probes like 5-aminolevulinic acid (5-ALA) or fluorescein sodium helps neurosurgeons distinguish tumor from normal brain tissue effectively in real-time despite neuronavigation and brain shifting, increasing resection success rates in malignant gliomas compared to conventional white-light resections—this pioneering technique, initially used for high-grade glioma (HGG) resection, has seen 5-ALA show a favorable safety profile, though its comprehensive advantages remain unestablished.^10,16–22^

The infiltrative characteristics of GBM pose a specific challenge because conventional imaging methods do not accurately define the boundary between the tumor and the brain or detect areas containing non-enhancing tumor cells.^1,23^ Recent advances in high-throughput sequencing have enhanced our capacity for molecular characterization of gliomas.^24^ The creation of surgical tools that facilitate the intraoperative detection of non-enhancing tumors could enhance cytoreductive procedures, potentially offering substantial benefits to the prognosis.^25–27^ Recent evidence suggests that the administration of fluorescence probes, such as 5-ALA or fluorescein sodium, allows for the intraoperative visualization of malignant glioma tissue. This technique offers immediate assistance to neurosurgeons in distinguishing between tumor and normal brain tissue in real time and remains effective despite neuronavigation and brain shifting. The use of 5-ALA-induced fluorescence has increased the success rate of resections in malignant gliomas compared to conventional white-light resections. Initially, this pioneering fluorescence technique was primarily employed in the resection of high-grade gliomas. While 5-ALA has shown a favorable safety profile, the comprehensive advantages linked to its application have not been definitively established.^22,28,29^

Surgically managing radiologically suspected GBM presents a distinct challenge for neurosurgeons due to the histopathological diversity and uncertain tumor boundaries.^12^ To achieve maximal resection safely, modern glioma surgery has adopted magnetic resonance imaging (MRI)-based neuronavigation as a standard approach. This combines precise spatial guidance, differential identification of tumor and non-tumor tissues, and neuroanatomical reference points.^26,30^ Despite its advantages, MRI-based neuronavigation faces challenges from inevitable brain shifts during surgery, the need for precise registration, and reliance on contrast enhancement to identify tumors. These factors can impede the real-time identification of resectable tumors, potentially leading to residual tumors at the resection margins.^4,31^ An interim analysis of a randomized trial comparing intraoperative MRI (iMRI)-guided glioblastoma (GBM) resection with conventional neuronavigation-guided resection did not demonstrate an advantage in terms of EOR, clinical outcomes, or survival for the iMRI group.^25^ However, a cohort study involving 135 patients with supratentorial GBM found that a combination of navigation and iMRI significantly achieved optimal EOR with low postoperative mortality. Notably, EOR ≥98% and patient age <65 years were associated with significant survival advantages. The median survival was 14 months for EOR ≥98% compared to 9 months for EOR <98% (*P* < 0.001).^32^ The use of iMRI increased EOR and the rate of gross total resection (GTR) and was a significant predictor of GTR according to multivariate analysis. However, GTR did not independently predict overall survival, and its use was not associated with an increased incidence of new permanent neurological deficits.^33^

A new technology that allows neurosurgeons to clearly visualize tumors and precisely differentiate them from normal brain tissue is highly desirable and could be a game-changer. The development of portable histopathological microscopy has enabled rapid and timely imaging of unprocessed tissue specimens. Following methylene blue staining, non-neoplastic tissues displayed well-organized glands consisting of regularly arranged cells. In regions of normal brain tissue, neurons with their axons and dendrites were evident. Conversely, in cancerous regions, nuclei exhibited irregular shapes and noticeable swelling, contrasting with those seen in non-neoplastic tissues. In this study, we successfully visualized cancer cells, normal brain cells, necrosis, and infiltrative cells in rat brains *in situ* during surgery. We also visualized cancer cells *in situ* from resected human brain samples from GBM patients. Data from this study demonstrate that the portable histopathological microscope can precisely localize positive surgical margins, enabling surgeons to accurately and safely resect tumors.

## RESULTS

### EndoSCell-guided glioma resection

**FIG. 1.**
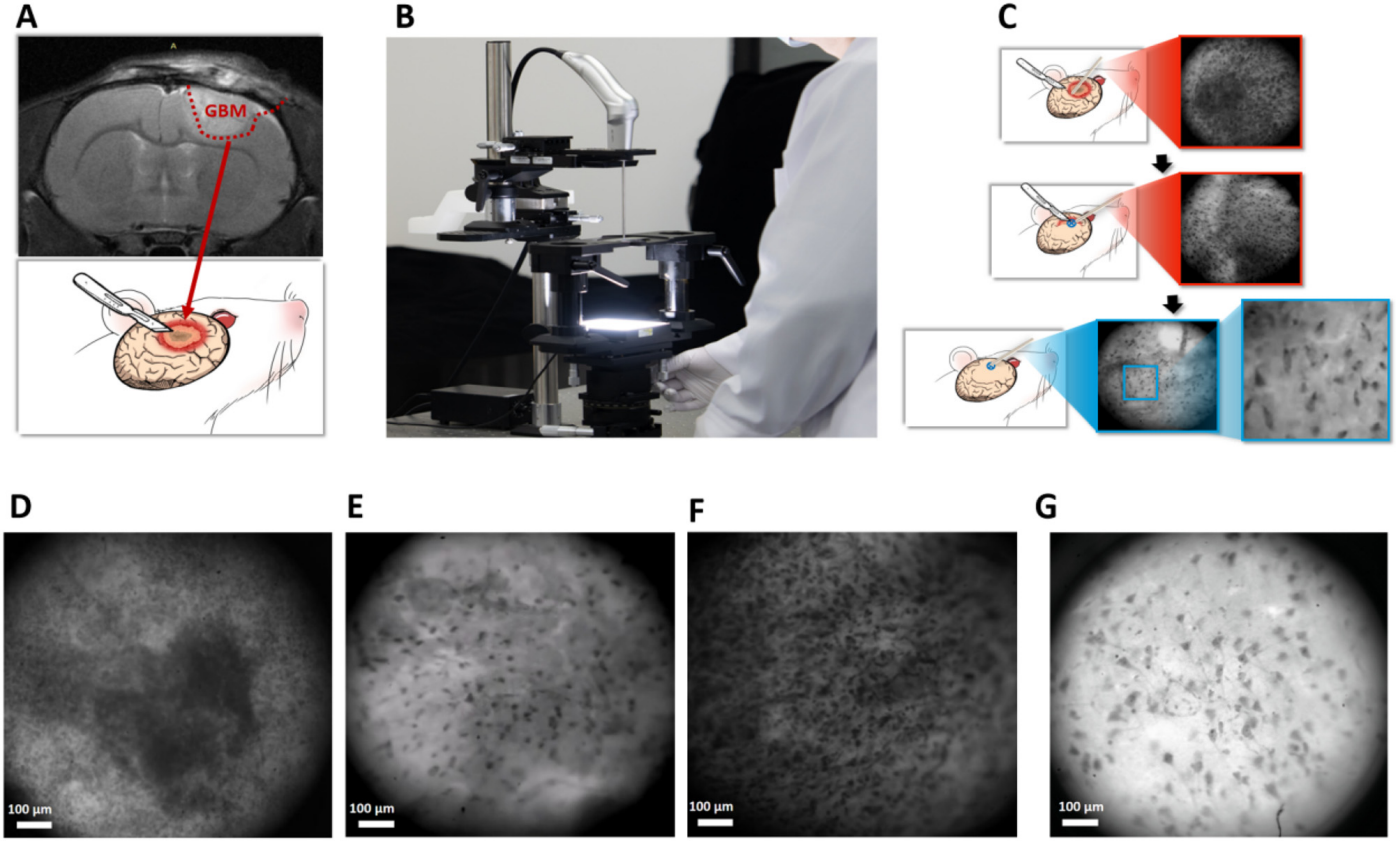
Schematic illustration of the portable histopathological microscopy system, EndoSCell. (a) In the white-light group, the tumor was meticulously removed until a negative margin was confirmed using conventional operative microscopy. (b) Tissue slices are displayed on a white surface, with a small microscope positioned over the illuminated area (the elongated metal rod represents the microscope). This miniature microscope facilitates *in vivo* observations, and the entire setup allows for clear visualization of the cellular structure on a computer screen. (c) Following the major debulking in (a), EndoSCell was employed using a “Find and Safe Removal Tactics” approach. The entire cavity was scanned to locate any positive margins, which were then refined until a safe negative margin was confirmed. (d) *In vivo* image of the tumor region, showing high cellularity with marked hyperchromatism and pleomorphism. (e) *In vivo* image from the EndoSCell-polished edge, displaying much lower cellularity, reduced hyperchromatism, and pleomorphism, with some cells showing characteristics of neuronal structures. (f) EndoSCell micrograph of resected brain tumor samples from the patient's tumor region, illustrating very high cellularity and deformed nuclei. (g) EndoSCell micrograph of extensively resected brain regions (gray matter), showing Nissl bodies and neuronal axons (blue arrow) as well as nucleoli (yellow arrow).

EndoSCell is an epifluorescence microscope utilizing dual contrast agents (sodium fluorescein and methylene blue) for surface staining. Its 475 nm LED excitation source (peak emission: 450–490 nm) targets sodium fluorescein's absorption profile. When applied to tissue, sodium fluorescein emits 530 nm green fluorescence upon excitation. Surgeons position the handheld probe directly against stained tissue surfaces, where the system's image acquisition module captures real-time cellular-level fluorescence. Processed signals are instantly displayed for intraoperative decision-making. This integrated workflow enables rapid, high-resolution imaging without disrupting surgical flow, a key advantage over bulkier conventional systems [Fig. 1(a)]. Compared to traditional white-light surgery using the naked eye or a stereoscope, this method enhances the ability to visualize and differentiate tissue structures [Fig. 1(b)]. To improve surgical precision, EndoSCell employed a rapid scan of the surgical area following tumor resection guided by MRI using the EndoSCell, identifying regions with heterogeneous shapes and swelling. Subsequently, a detailed scan of the areas marked with an asterisk was conducted to further refine the resection [Fig. 1(c)]. Thus, it facilitates the differentiation between tumor and normal brain tissue. In cancer tissue, a glioblastoma xenograft shows increased nuclear density and varying staining, size, and shape of nuclei [Fig. 1(d)]. In contrast, normal brain tissue is characterized by one or two layers of glial cells or neurons, with a low nuclear-to-cytoplasm ratio and consistent nuclear sizes [Fig. 1(e)]. The miniaturized microscope facilitates the intraoperative localization of residual tumor regions. The EndoSCell micrograph provides an *ex vivo* depiction of resected brain tumor samples obtained from the patient's tumor region, illustrating significantly elevated cellularity and the presence of deformed nuclei [Fig. 1(f)]. Additionally, another micrograph shows an *ex vivo* view of extensively resected brain regions, specifically the gray matter, where distinct features such as Nissl bodies and neuronal axons, as well as nucleoli, are observed [Fig. 1(g)]. These observations offer valuable insights into the cellular structures and composition of the resected brain tissue, enhancing the overall understanding of the surgical outcomes and tissue integrity.

### Precise resection but not extended resection contributes to prognosis

**FIG. 2.**
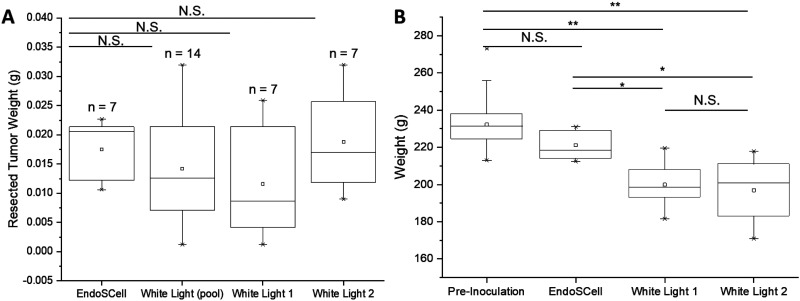
Tumor resected weight and body weight comparison after surgery. (a) The bar graph shows that there was no significant difference in tumor weight between the EndoSCell-surgery group and the white-light surgery groups. (b) Body weight comparison before inoculation and 10 days post-surgery. Rats treated with EndoSCell-guided surgery maintained body weight comparable to the pre-inoculation group, whereas rats in the white-light surgery groups showed a notable decrease in body weight (N.S., not significant; ^*^P < 0.05; ^**^P < 0.01).

During the procedural process, no significant subjective differences were noted among the three groups. To mitigate potential subjectivity introduced by the surgeons, the weight of resected tumor tissue was measured and compared. Statistical analysis revealed no significant differences between the groups (Fig. 2), suggesting that no surgeon performed extended resections. Therefore, any variations in prognosis are likely due to differences in the precision and standardization of the resection technique rather than extended resection. Additionally, comparison of rat body weights [Fig. 2(b)] before tumor inoculation and 10 days post-surgery revealed that animals in the EndoSCell-guided surgery group maintained body weight levels comparable to their pre-inoculation state. In contrast, rats in the white-light surgery groups experienced substantial weight loss, with white light 1 and white light 2 groups showing average reductions of 15.3% and 13.9%, respectively. These findings suggest that conventional white light-guided surgery may lead to greater postoperative burden and compromised health status, whereas EndoSCell-guided surgery better preserves systemic health and recovery.

### Prognosis of EndoSCell-guided surgery

**FIG. 3.**
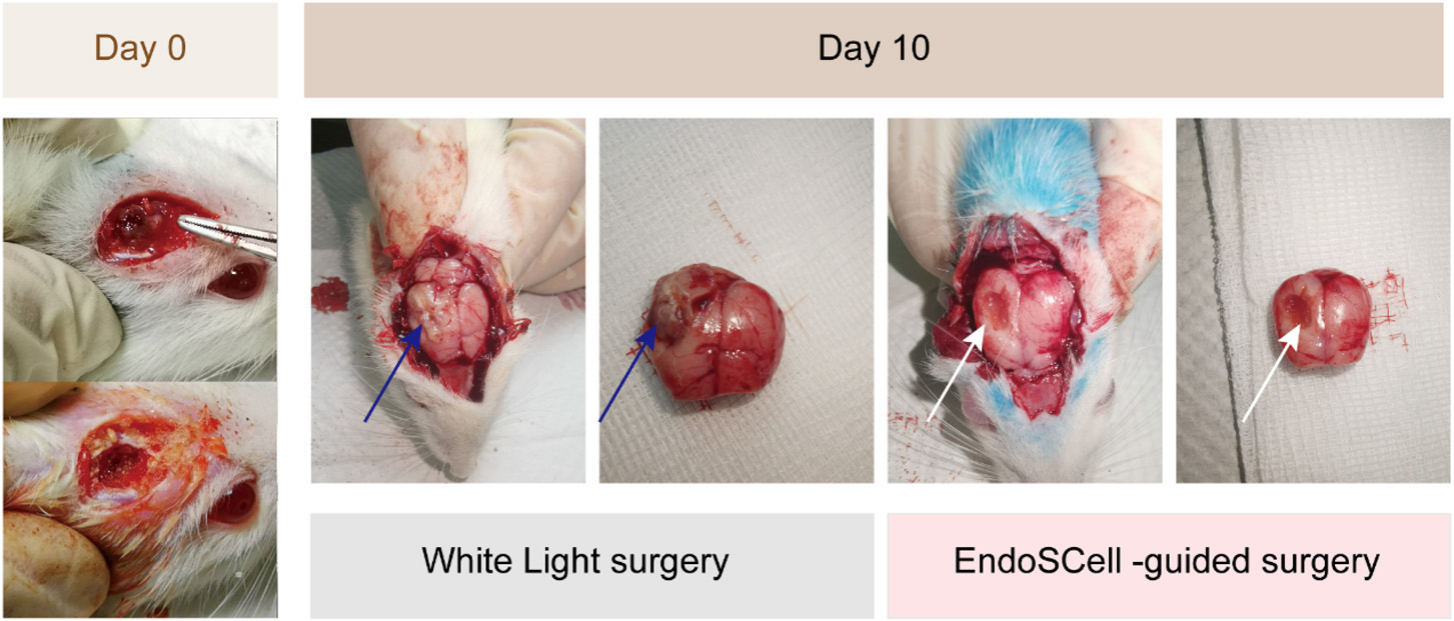
Gross appearance of postoperative brain parenchyma. At day 0, the brain cavity post-surgery is visible. By day 10, the EndoSCell group showed no apparent tumor in the resected cavity (white arrow), with the cavity edges coated with Floseal. Conversely, in the white-light surgery group, the resected cavity was filled with gray, soft, necrotic brain tissue, and the tumor (blue arrow) had rapidly grown, extending beyond the brain surface.

To evaluate prognosis, contrast-enhanced MRI scans were performed after administering a paramagnetic contrast agent. MRI images of the rat brain were captured pre-surgery and at specific intervals postoperation. Initial tumor xenograft volumes ranged from 60 to 120 × 10^−3^ mm^3^ and were primarily located in the ipsilateral cortex. The mean tumor volumes were 86 ± 25.74 × 10^−3^ mm^3^ in the white-light surgery group 1, 98 ± 24.69 × 10^−3^ mm^3^ in white-light surgery group 2, 90 ± 30.78 × 10^−3^ mm^3^ in EndoSCell-assisted surgery group 3, and 88 ± 28.92 × 10^−3^ mm^3^ in the sham procedure group. These differences were statistically nonsignificant (*P* = 0.437) (Fig. 3). Tumor infiltration into adjacent striatum and hippocampus was also observed. These results suggested that, from an anatomical perspective, tumor recurrence was notably observed in rats subjected to conventional white-light surgery, whereas no recurrence was detected in rats undergoing surgery guided by EndoSCell technology.

**FIG. 4.**
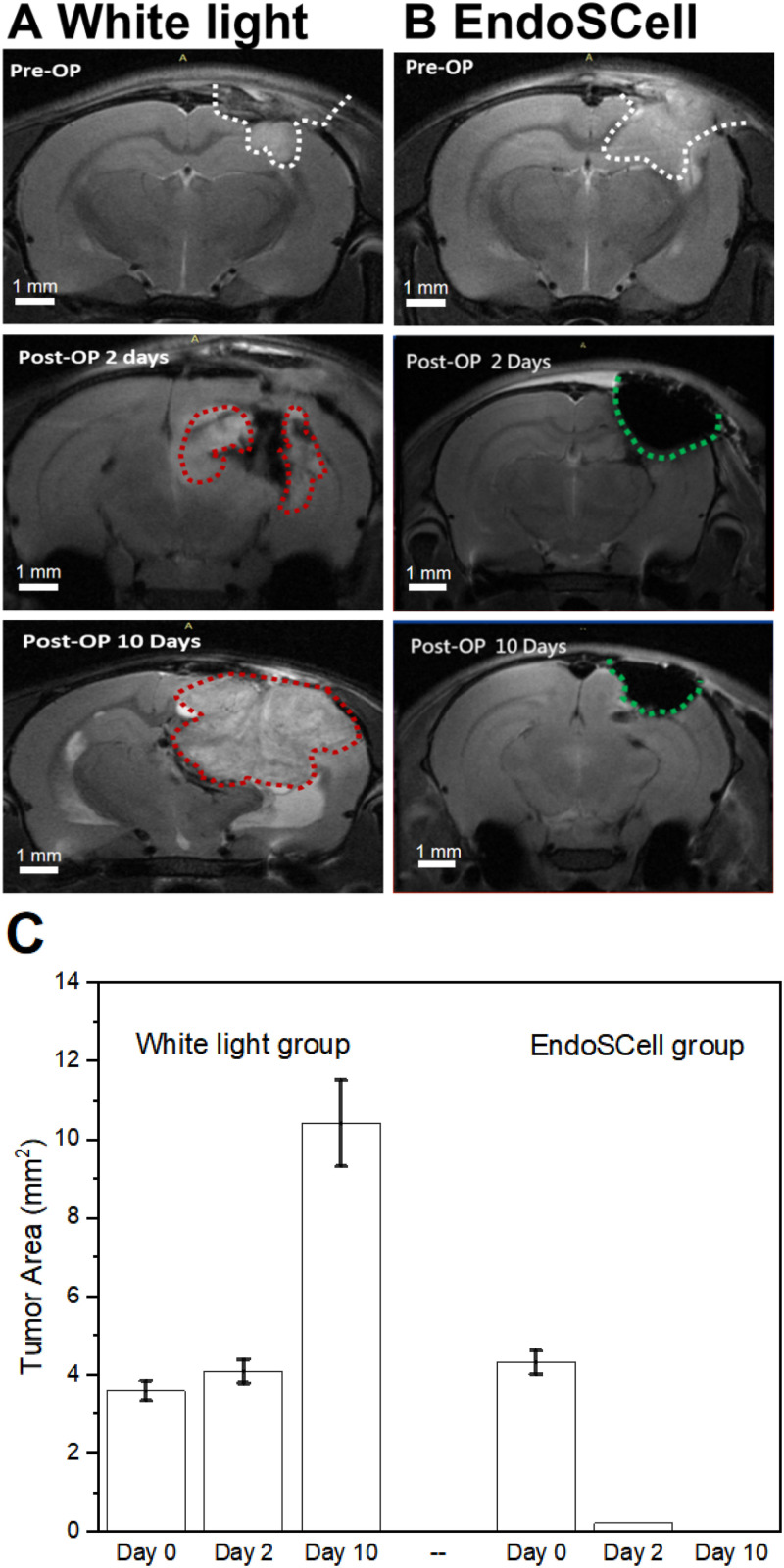
MRI evaluation of tumor recurrence after surgical resection. Representative coronal MRI images illustrate tumor progression in two groups across three time points: preoperation, postoperative day 2, and postoperative day 10. (a) In the white light-guided surgery group, residual tumor is visible as early as day 2 and shows substantial regrowth by day 10, as outlined by red dotted lines. (b) In contrast, the EndoSCell-guided surgery group exhibits complete tumor resection with no detectable recurrence at either postoperative time point, as indicated by green dotted lines. (c) Quantitative analysis of tumor area confirms the effectiveness of EndoSCell-guided resection in achieving complete removal and preventing recurrence. A reduction in postoperative tumor area is observed in the EndoSCell group, whereas the white-light group shows marked tumor regrowth.

To assess tumor removal and recurrence, contrast-enhanced T2-weighted MRI was used at postoperative days 2 and 10. In the EndoSCell-guided surgery group, only minimal MRI signal enhancement was observed adjacent to the resection cavity on day 2, which did not increase by day 10. Remarkably, only one out of seven rats (14%) in this group exhibited tumor recurrence, as shown in Fig. 4(a). In contrast, the white light-guided surgery group demonstrated a prominent MRI signal within the tumor bed as early as day 2, which markedly expanded in both intensity and area by day 10. By this time point, 12 out of 15 rats (80%) exhibited clear evidence of tumor recurrence on MRI scans [Fig. 4(b)].

Quantitative analysis of tumor area [Fig. 4(c)] further substantiated the therapeutic benefit of EndoSCell-guided surgery. In the white-light group, tumor area increased significantly from 3.58 ± 0.2 mm^2^ on day 0 to 10.4 ± 1.1 mm^2^ on day 10, representing an approximately 290 ± 7.4% larger tumor area. This growth correlated with observable neurological symptoms, including lethargy, reduced appetite, and progressive weight loss, aligning with the body weight data in Fig. 2(b). Notably, these animals also exhibited a marked increase in mortality within 2 weeks post-surgery, likely due to tumor-induced compression of vital brain regions. In contrast, the EndoSCell group showed dramatic tumor regression, with tumor area decreasing from 4.3 ± 0.3 mm^2^ to undetectable levels by day 10. Even the initial resection cavity area shrank significantly, from 4.31 ± 0.3 to 2.2 ± 0.1 mm^2^ within 10 days, indicative of effective surgical clearance and robust postoperative recovery. These results highlight the superior precision and clinical potential of EndoSCell-guided surgery in minimizing residual tumor burden and enhancing brain tissue healing.

### Histological analysis of tumor recurrence

**FIG. 5.**
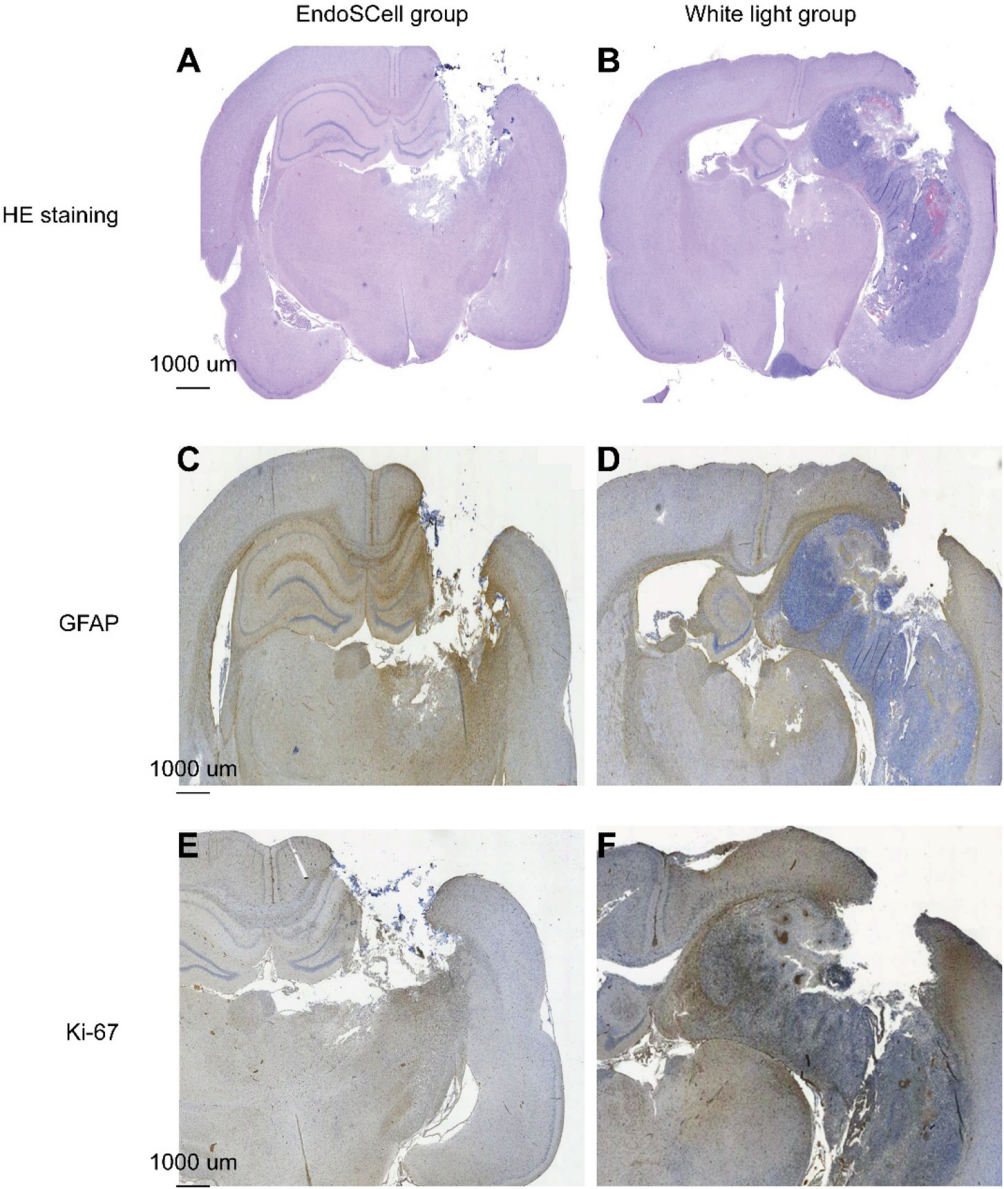
Histological and immunohistochemical analysis of tumor recurrence. (a) and (b) H&E staining shows no obvious karyorrhectic cells in the EndoSCell group, while diffuse karyorrhectic tumor cells with central necrosis confirm tumor recurrence in rats undergoing white-light surgery. (c) and (d) GFAP immunohistochemistry staining reveals normal GFAP-positive glial cells surrounding the resected cavity in the EndoSCell group and a large area of GFAP-negative glial cells in the white-light surgery group. (e) and (f) Ki-67 immunostaining demonstrates numerous Ki-67-positive cells at the edges of the resected cavity in the white-light group, with no Ki-67-positive cells observed in rats undergoing EndoSCell-assisted surgery.

To confirm tumor recurrence, histological analysis was performed using hematoxylin and eosin (H&E) staining and immunohistochemistry with glial fibrillary acidic protein (GFAP) and Ki-67 markers. H&E staining results revealed a significant tumor presence in the white-light surgery group, characterized by diffusely karyorrhectic glioma cells and coagulative necrosis, while the EndoSCell group exhibited no obvious tumor area [Figs. 5(a) and 5(b)]. Immunocytochemical staining for GFAP showed GFAP-positive immunoreactivity at the periphery of the resected tumor cavity, with a substantial area of GFAP-negative cells (blue) along the tumor cavity [Figs. 5(c) and 5(d)]. The Ki-67 labeling index indicated no Ki-67-positive regions in the EndoSCell-guided surgery group, whereas numerous dark-brown Ki-67-positive cells were observed around the margin of the resected tumor in the white-light surgery group [Figs. 5(e) and 5(f)].

### Survival rate of rats following white light- or EndoSCell-guided surgery

**FIG. 6.**
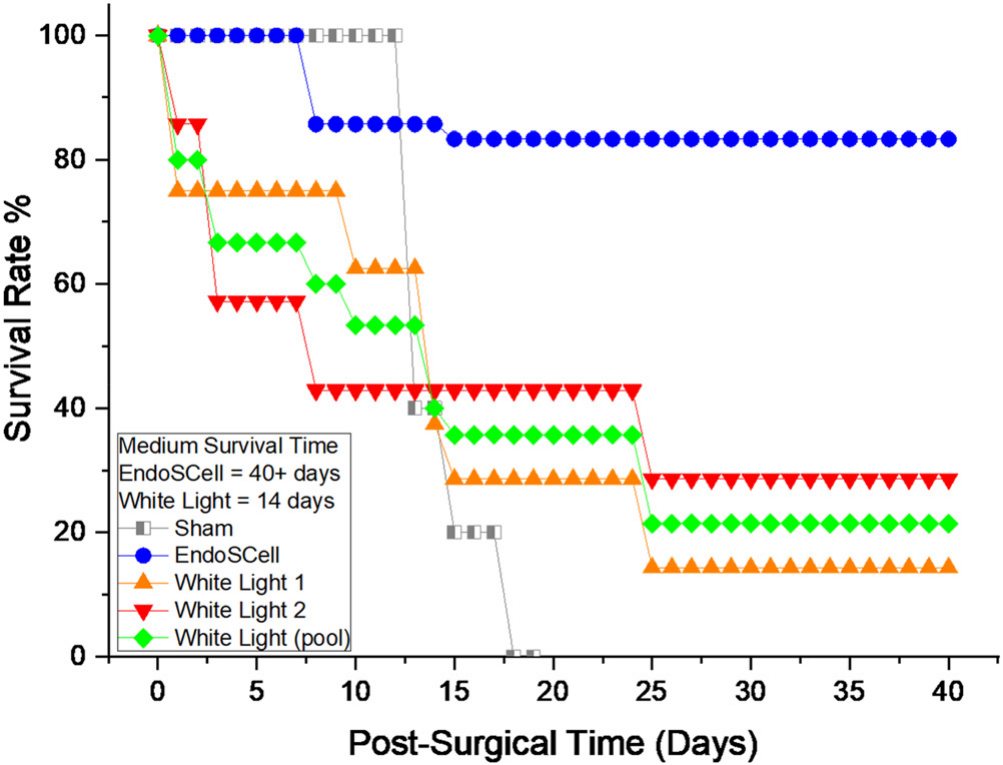
The survival rates of rats were significantly extended by EndoSCell-guided surgery in glioma-bearing rats. The difference between the EndoSCell group and the conventional white-light surgery group reached statistical significance (^***^P < 0.001).

The survival rate of rats showed a remarkable improvement with EndoSCell-guided surgery. In the EndoSCell-guided surgery group, six out of seven rats survived for 40 days, resulting in an 86% survival rate post-surgery. In contrast, only 3 out of 15 rats (20%) in the conventional white-light surgery group survived the same period. These findings strongly suggest that EndoSCell-guided surgery not only helps achieve negative margins but also significantly reduces tumor recurrence, leading to a substantial increase in survival rates (86% vs 20%) (*P* < 0.001) (Fig. 6).

## DISCUSSION AND CONCLUSION

Surgical resection is an effective means of reducing tumor size and mass. However, the highly aggressive, infiltrating nature, rapid growth, and diffuse recurrence of GBM invariably result in fatality for patients.^35^ The influence of the EOR on the survival of GBM patients remains a topic of contention, despite several studies illustrating that maximal tumor removal likely prolongs survival, particularly among younger individuals.^8,11,13,36^ However, overly aggressive resections may not benefit patients, as they can result in injuries to non-regenerative nerves, vessels, or intricate areas that control consciousness, movement, or other vital functions.^15,20^ Precise intraoperative and real-time tissue diagnosis plays a pivotal role in enhancing surgical outcomes. Currently, neurosurgeons and pathologists employ diagnostic techniques such as frozen sectioning and smear preparations. While these methods are reliable, they require lengthy preparation and testing times, which can impact the diagnostic process. Therefore, there is a need for a simplified and efficient histological testing approach that can significantly reduce the testing time.^3,7,18^

Current intraoperative histologic methods, such as frozen sectioning and cytologic preparations, require the expertise of proficient technicians and clinicians in surgical pathology labs to generate and analyze slides. Unfortunately, the volume of brain tumor surgeries far surpasses the available capacity of board-certified neuropathologists, often precluding the option of obtaining expert intraoperative guidance in many cases. Even within the most advanced and adequately staffed healthcare institutions, the time required for intraoperative pathology reports can delay clinical decision-making during surgery. This further underscores the necessity of studying new systems.^3,18,23,37^ Furthermore, given that the percentage of tumor removed during surgery is a significant prognostic factor for brain tumor patients, the use of intraoperative techniques to accurately identify residual tumor is essential.

The use of 5-ALA fluorescence-guided surgery has shown an increased success rate in achieving total resection of contrast-enhancing tumor tissue, leading to significantly prolonged progression-free survival compared to white-light surgery.^20,28^ Consequently, 5-ALA received approval for HGG resections in the European Union in 2007. The utilization of 5-ALA fluorescence-guided surgery has since gained popularity in neurosurgery due to its cost-effectiveness, widespread availability, and minimal side effects. Nonetheless, the current 5-ALA technique is constrained by the frequent absence of visible fluorescence in pure gliomas.^38^ A blinded, controlled multicenter phase III trial was launched to examine the effects of 5-ALA fluorescence-guided resection on the EOR and progression-free survival in high-grade glioma (HGG). Unfortunately, 5-ALA fluorescence is typically detectable only in HGG and is generally not observable in most low-grade gliomas or early infiltrative regions.^16,31,39^ A nationwide, multicenter, prospective observational study indicated that 5-ALA may not be considered a critical tool for HGG resection due to its less-than-optimal ability to differentiate between normal and tumor cerebral tissue using fluorescence.^22^

Using information gathered from a randomized controlled multicenter phase III trial, it was observed that patients with HGG who underwent 5-ALA fluorescence-guided surgery exhibited better rates of achieving complete resections and experienced enhanced progression-free survival compared to those who underwent conventional operative microscopy.^10^ 5-ALA assists in distinguishing between normal and malignant tumor tissue, enabling real-time image-guided surgery that has the potential to augment the EOR by 24.5% (47.4% vs 22.9%).^7,40^ However, some critics have reported that 5-ALA may not be sensitive enough to achieve optimal prognosis.^21,38^ A more recent approach to GBM surgery utilizes protoporphyrin IX (PpIX) and 5-ALA, but it has demonstrated limitations in detecting grade 2 gliomas and invasive cancer cells.^4,41^ An *ex vivo* neuropathological analysis revealed that a substantial percentage of biopsy sites (∼40%) were positive for tumors but did not exhibit visible fluorescence in both low- and high-grade gliomas. This suggests a significant accumulation of PpIX below the detection threshold of current fluorescence imaging techniques.^18,19^

Other technologies have been developed to enable real-time cellular-level optical biopsies, including Endocyto (Olympus) and the Confocal Laser Endomicroscope (CLE). Endocyto, a semi-transmission microscope with an 11 mm diameter, is typically employed for histopathological diagnosis through natural orifices in the gastrointestinal (GI) tract due to its relatively higher invasiveness.^37,42^ However, neurological surgeries are more sensitive to invasiveness, making a smaller diameter probe more favorable.

Surgical margin investigation requires high imaging resolution to observe organelles, such as the nucleus and nucleolus, which are normally sub-micron scale. Thus, outstanding optical performance with sub-cellular, sub-micron resolution is crucial for diagnostic sensitivity and accuracy. *In vivo* intraoperative imaging is highly sensitive to motion artifacts from respiration, heartbeats, and handshakes from surgeons holding the device. Previous studies have reported that other technologies, such as CLE, require a large number of images to select a few high-quality ones due to lower image quality and sensitivity to vibrations. Two clinically approved CLE systems currently in use are the CellVizio from MKT and the ISC-1000 from Pentax/Optiscan. Obtaining movement-free confocal images is a recognized challenge in CLE, especially when dealing with pedunculated lesions or those positioned tangentially to the confocal scanning window. Intravenous administration of a smooth muscle relaxant before CLE image capture, along with gentle suction and/or the use of an endoscopic cap, has proven effective in stabilizing the equipment and reducing excessive movement. However, these stabilizing methods may not be applicable in all scenarios, such as glioma resection.^37,42,43^

EndoSCell can now be utilized in a clinical environment to promptly evaluate tissue architecture, with minimal disruption to the surgical process. The images acquired using EndoSCell have the potential to support highly precise diagnoses of brain tumor specimens, aligning closely with established intraoperative histologic methods. To validate these findings and determine the potential impact of EndoSCell on expediting clinical decision-making and enhancing the care of brain tumor patients, prospective randomized clinical trials will be essential. Utilizing glioma xenografts in rats, EndoSCell effectively delineated the invasive tumor margin with a high level of precision. Initially, preoperative MRI was utilized to establish the location of orthotopic glioma xenografts in the rat brain, guiding the surgical craniotomy plan. Remarkably, MRI results demonstrated that EndoSCell-guided surgery significantly decreased the rate of tumor recurrence and enhanced the overall survival of rat models in comparison to alternative techniques. Moreover, the portable and lightweight design of EndoSCell allowed it to reach deep tumor cavities, enabling more precise resections. iMRI is another advanced tool used to guide tumor resection. However, iMRI possesses significant limitations that hinder its ability to provide the real-time, cellular-level information critical for precise margin delineation. First, iMRI operates at a macroscopic anatomical level, lacking the cellular-level resolution necessary to visualize microscopic tumor boundaries. Second, image acquisition requires interrupting the surgical workflow, preventing real-time feedback during resection. Furthermore, iMRI systems are bulky, operationally complex, require specialized MRI-compatible operating rooms, and incur prohibitively high per-procedure costs. These limitations underscore the need for alternative intraoperative imaging solutions capable of providing high-resolution, real-time cellular visualization without disrupting surgery.

Overall, this study highlights the prognostic advantages of using EndoSCell for glioma surgery in animal models. Given the common challenge surgeons face in delineating tumor-invasive margins, EndoSCell holds promise for improving surgical outcomes in various types of infiltrative tumors. Furthermore, it enhances safety, as incomplete resections can lead to general motor dysfunction and worsen overall health. Beyond identifying tumor regions, EndoSCell can help identify safe areas, particularly vital for tumors located near critical life-regulating regions such as the brainstem. This capability can prevent unintentional damage to these functional areas, preserving function and enhancing patients' quality of life.

Both GBM and astrocytoma originate from astroglial cells, and the number of cells expressing GFAP typically decreases as the degree of anaplasia increases. In high-grade astrocytoma, the loss of GFAP expression is frequently observed.^44^ GFAP-positive glial cells were established along the margin of the resected tumor cavity in the EndoSCell-assisted group, and only a few GFAP-positive cells were detected at the margin of the resected tumor cavity in the white-light surgery group. Numerous and densely Ki-67-labeled cells accumulated around the margin of the resected tumor cavity in the white-light group but were much lower in the EndoSCell group. In this study, 9L xenograft animal models were established using healthy rats, and the experimental results demonstrated that EndoSCell was useful for identifying tumor cells and normal glial cells intraoperatively, achieving accurate resection of tumors with the preservation of healthy brain tissue. Precise resection of MG exhibited significant therapeutic efficacy, with extended survival and reduced recurrence of MG. However, the absence of postoperative MRI volumetric analysis constitutes a limitation in the precise evaluation of resection margins, as assessment relied on resected tumor weight rather than spatial volumetric data.

The clinical implementation of the EndoSCell-guided glioma surgery approach still encounters challenges. First, EndoSCell imaging alone cannot visualize gross tumors adequately due to its limited biological penetration. Thus, whole-body imaging methods, particularly MRI, are important for the primary localization of gliomas. Furthermore, by utilizing a device capable of accurately and easily visualizing distinctions related to glioma malignancy, genotype, and phenotype, it becomes possible to identify the invasive regions of gliomas. EndoSCell can provide high-resolution, noninvasive imaging, enabling differentiation between glioma cells and normal brain cells. Real-time identification of tumor margins in EndoSCell imaging could be advantageous for both surgeons and patients, facilitating safe maximal resection, minimizing the removal of excess normal brain tissue, and ultimately extending survival rates. In the future, EndoSCell holds great promise for carrying out a multicenter clinical trial to examine its usefulness in assisting surgery and its impact on patient prognosis.

## METHODS

### Experimental equipment

The experimental setup utilized for this study consists of a portable histopathological microscopy system known as the EndoSCell, developed by Dendrite Precision. The study utilized 26 healthy rats, allocated as follows: group 1 (white-light surgery 1, n = 7), group 2 (white-light surgery 2, n = 7), group 3 (EndoSCell-guided surgery, n = 7), and group 4 (sham operation, n = 5). The EndoSCell is a miniaturized epifluorescence microscope that provides optical performance equivalent to that of a traditional histopathological microscope, with comparable field of view and lateral resolution. The EndoSCell exhibits a penetration depth of 100 *μ*m, allowing observation of four to five cell layers within a relatively expansive field of view measuring 500 *μ*m.^34^ It operates at a rapid frame rate of 60 Hz, delivering exceptionally stable real-time images from the surgical margin, thanks to its autofocus capability. With these features, the EndoSCell proves to be an ideal tool for examining the tumor microenvironment, promising valuable insights into this critical aspect of histopathological research.

### Prognostic value of an EndoSCell-assisted surgery

We enrolled 26 glioma-bearing rats and randomly divided them into four groups: group 1, group 2, group 3, and group 4. Groups 1 and 2 included rats that underwent craniotomies for tumor removal, with surgeries in group 1 performed by surgeon 1 and in group 2 by surgeon 2, both using conventional white-light microscopy to examine tumor margins. Group 3 comprised seven rats randomly selected from groups 1 and 2 (three from group 1 and four from group 2), where craniotomies were performed similarly but tumor margins were examined using EndoSCell. Group 4, the sham group, consisted of five rats that did not undergo any surgical procedure. All rats, including those in the sham group, underwent comprehensive analyses, including MRI, pathological examination, and survival analysis.

The animals were anesthetized with Rompun 20 and Zoletil (Virbac), followed by Isoflurane, and then positioned on the operating table. The craniotomy was performed in a stepwise manner: first, the skin was incised, the muscle was dissected, and the skull was drilled. A 1.5 × 1.5 cm skull defect was created based on preoperative MRI findings. The dura mater was carefully removed in a curved fashion, with the dural flap oriented toward the superior sagittal sinus.

### Surgical procedures

In the *in vivo* test, orthotopic 9L glioblastoma xenografts were randomly assigned to three groups, each undergoing either EndoSCell-guided surgery or conventional white-light surgery. Prior to surgery, all models underwent T1- and T2-weighted MRI scans to accurately locate the tumor. The xenografts were primarily situated in the ipsilateral striatum, with infiltrative edges extending into the adjacent hippocampus and cortex. MRI imaging guided all three surgical approaches.

Based on the tumor's location as defined by preoperative MRI, a craniotomy was performed in the right frontoparietal region near the bregma. The surgical procedure involved skin and muscle incision, followed by skull drilling. A 2.0 × 1.5 cm skull window was created, and the dura mater was carefully removed in a curved fashion, with the base aligned along the superior sagittal sinus to avoid damaging underlying blood vessels. In conventional white-light surgery, tumor resection was primarily guided by the surgeon's experience and the observable characteristics of the tumor from preoperative MRI. A thorough examination of the tumor margins was conducted using conventional operative microscopy to ensure they were free of tumor tissue.

### *In vivo* observation

Methylene blue (1%) was locally perfused into the cavity for 2 min for *in vivo* staining. Following surgical removal, cell morphology and cellular patterns within the intracranial xenograft cavity were observed using EndoSCell. All images were jointly evaluated by an endoscopist and a pathologist. Subsequently, lesions were histologically diagnosed based on biopsy specimens.

### Establishment of orthotopic glioblastoma xenograft

#### Establishment of a rat tumor model

The 9L rat glioma cell line was purchased from the American Type Culture Collection and maintained through regular subculturing. Cells were cultured at 37 °C with 5% CO_2_ in Dulbecco's modified Eagle's medium supplemented with 10% fetal bovine serum, penicillin (100 U/ml), and streptomycin (100 *μ*g/ml). Female Sprague Dawley rats, aged 5–7 weeks and weighing approximately 250 g, were obtained from the National Laboratory Animal Center and housed under controlled conditions. For the experiments, the rats were anesthetized with Rompun 20 and Zoletil (Virbac), followed by Isoflurane. To induce tumors, 9L glioblastoma cells (5 × 10^5^ cells in 2 *μ*l phosphate-buffered saline) were stereotaxically injected into the right striatum, adjacent to the bregma, to a depth of 5.0 mm using a 25 *μ*l syringe. The injection was performed at a rate of 2.0 *μ*l/min, with the syringe remaining in place for an additional 3 min after injection completion. Tumor presence was confirmed by T2-weighted MRI, which was conducted 10 days post-inoculation, as well as 2 days before surgery and at 2, 10, and 17 days following the surgical procedure.

#### Establishment of patient-derived tumor xenograft

Glioma stem cells, derived from patients, were cultured in Neurobasal medium supplemented with B27 (lacking Vitamin A, Invitrogen), basic fibroblast growth factor (bFGF, 20 ng/ml, R&D Systems), and epidermal growth factor (EGF, 20 ng/ml, R&D Systems). The uniqueness of each specimen was validated through short tandem repeat analysis. Routine screenings for mycoplasma contamination were conducted on all cell lines, consistently yielding negative results.

For intracranial xenograft implantation, cells were introduced into NOD SCID mice of both genders, aged 4–6 weeks. Neurological symptoms served as the end point for these experiments. In concurrent survival studies, rats were monitored continuously until the onset of neurological symptoms. To perform hematoxylin and eosin (H&E) staining, two rats from each group were euthanized 2 weeks after the intracranial injection.

### *Ex vivo* human sample observation from GBM patients

Tumor tissues from GBM patients were used for *in situ* EndoSCell observation. Imaging was performed on freshly resected tumor samples after 2 min of methylene blue staining to simulate *in vivo* intraoperative conditions. Following EndoSCell imaging, the same tissues were sent for frozen sectioning and pathological examination.

### Statistics

Statistical measures are expressed as means ± standard deviation. Analyses were performed using SPSS version 19.0 for Windows (SPSS Inc., USA). Comparisons were conducted with the Student's *t*-test, and a two-tailed *P*-value of <0.05 was considered statistically significant.

## Data Availability

The data that support the findings of this study are available from the corresponding author upon reasonable request.
